# Supplementary material for: Allele-specific miRNA-binding analysis identifies candidate target genes for breast cancer risk
Source: NPJ Genom Med. 2020 Feb 13;5:4. doi: 10.1038/s41525-019-0112-9 (PMC7018948; doi:10.1038/s41525-019-0112-9)
Supplement: Supplementary file 1 — Supplementary Information [file 41525_2019_112_MOESM1_ESM.pdf]

Supplementary Information

Allele-specific miRNA-binding analysis identifies candidate target genes for breast cancer risk

Ana Jacinta-Fernandes[1,2,3], Joana M. Xavier[1,2,3], Ramiro Magno[2,3], Joel G. Lage[1,3] and Ana-Teresa Maia[1,2,3*]

[1] Department of Biomedical Sciences and Medicine (DCBM), Universidade do Algarve, Campus de Gambelas, 8005-139 Faro, Portugal

[2] Centre for Biomedical Research (CBMR), Universidade do Algarve, Campus de Gambelas, 8005-139 Faro, Portugal.

[3] Algarve Biomedical Center (ABC), Universidade do Algarve, Campus de Gambelas, 8005-139 Faro, Portugal.

Table S1. 150 variants previously associated with breast cancer risk.

SNP[1]	Locus	Position[2]	Allele[3]	AA[4]	MA[5]	Mapped Gene	Context	RAF[6]	*P*	OR [95% CI][7]	Trait[8]	Ref
rs186507655	1p36.33	1351675	G/A	G	A	*DVL1 - MXRA8*	upstream gene variant	–	5×10^{-10}	–	Cancer (pleiotropy)	[1]
rs616488	1p36.22	10506158	G/A	A	G	*PEX14*	intron variant	0.665	1×10^{-08}	1.10 [1.06-1.14]	BC	[2]
								0.670	2×10^{-10}	1.06 [1.04-1.09]	BC	[3]
rs12118297	1p22.3	87313534	T/G	G	T	*LOC101927844 - LMO4*	intergenic variant	0.620	4×10^{-08}	1.10 [1.06-1.14]	BC	[4]
rs11552449	1p13.2	113905767	C/G/T	C	T	*DCLRE1B*	missense variant	0.170	2×10^{-08}	1.07 [1.04-1.09]	BC	[3]
rs11249433	1p11.2	121538815	A/G	A	G	*EMBP1*	intron variant	0.390	7×10^{-10}	1.16 [1.09-1.24] (Het)	BC	[5]
								0.400	2×10^{-26}	1.09 [1.07-1.11]	BC	[3]
rs12405132*	1q21.1	145790097	A/G	G	A	*RNF115*	intron variant	0.640	8×10^{-09}	1.05 [1.03-1.08]	BC	[6]
rs12048493*	1q21.2	149955122	A/C	A	C	*OTUD7B*	intron variant	0.340	1×10^{-09}	1.07 [1.05-1.10]	BC	[6]
rs6678914	1q32.1	202218048	A/G	A	A	*LGR6*	intron variant	0.590	1×10^{-08}	1.10 [1.06-1.13]	BC	[2]
rs4951011	1q32.1	203797203	A/G	A	G	*ZC3H11A*	5’ UTR variant	0.282	9×10^{-09}	1.09 [1.06-1.12]	BC	[7]
rs4245739	1q32.1	204549714	A/C	A	C	*MDM4*	3’ UTR variant	0.260	2×10^{-12}	1.14 [1.10-1.18]	BC	[2]
rs72755295*	1q43	241870961	A/G	A	G	*EXO1*	intron variant	0.030	2×10^{-08}	1.15 [1.09-1.22]	BC	[6]
rs12710696	2p24.1	19121042	C/T	C	T	*LOC105373455 - MIR4757*	intron variant	0.360	5×10^{-08}	1.10 [1.06-1.13]	BC	[2]
rs4849887	2q14.2	120487546	T/C	T	T	*LINC01101 - LOC105373585*	intergenic variant	0.902	4×10^{-11}	1.10 [1.06-1.14]	BC	[3]
rs2016394	2q31.1	172108243	A/G	G	A	*DLX2-AS1*	intron variant	0.520	1×10^{-08}	1.05 [1.03-1.08]	BC	[3]
rs1550623	2q31.1	173348166	G/A	A	G	*LOC100289479 - CDCA7*	intron variant	0.840	3×10^{-08}	1.06 [1.03-1.09]	BC	[3]
rs13393577	2q34	212432139	C/T	T	C	*ERBB4*	intron variant	0.051	9×10^{-14}	1.53 [1.37-1.70]	BC	[8]
rs13387042	2q35	217041109	G/A	G	A	*LOC105373874, LOC101928278*	intergenic variant	0.500	1×10^{-13}	1.20 [1.14-1.26]	BC	[9]
								0.510	2×10^{-08}	1.25 [1.15-1.37] (Het)	BC	[10]
								0.520	2×10^{-10}	1.16 [1.11-1.22]	BC	[11]
								0.510	2×10^{-57}	1.14 [1.11-1.16]	BC	[3]
								0.490	2×10^{-10}	1.21 [1.14-1.29]	BC	[12]
rs16857609	2q35	217431785	C/T	C	T	*DIRC3*	intron variant	0.260	1×10^{-15}	1.08 [1.06-1.10]	BC	[3]
rs6762644	3p26.1	4700592	A/G	A	G	*ITPR1*	intron variant	0.400	2×10^{-12}	1.07 [1.04-1.09]	BC	[3]
rs481519	3p24.1	27285723	C/T	T	C	*NEK10*	intron variant	–	2×10^{-09}	–	Cancer (pleiotropy)	[1]
rs653465	3p24.1	27302153	C/T	T	C	*NEK10*	intron variant	0.540	5×10^{-12}	1.18 [1.12-1.23]	BC (early onset)	[13]
rs4973768	3p24.1	27374522	T/C	T	T	*SLC4A7*	3’ UTR variant	0.490	2×10^{-08}	1.14 [1.09-1.19]	BC	[11]
			C/T					0.470	2×10^{-30}	1.10 [1.08-1.12]	BC	[3]
rs12493607	3p24.1	30641447	G/T/C	C	C	*TGFBR2*	intron variant	0.350	2×10^{-08}	1.06 [1.03-1.08]	BC	[3]
rs6796502*	3p21.31	46825376	A/G	G	A	*PRSS43 - PRSS42*	downstream gene variant	0.910	2×10^{-08}	1.09 [1.05-1.12]	BC	[6]
rs1053338*	3p14.1	63982224	A/G	A	G	*ATXN7*	missense variant	0.130	9×10^{-09}	1.08 [1.05-1.11]	BC	[6]
rs7679673	4q24	105140377	C/A	A	C	*LOC100288146 - TET2*	intron variant	–	1×10^{-10}	–	Cancer (pleiotropy)	[1]
rs9790517	4q24	105163621	C/T	C	T	*TET2*	intron variant	0.230	4×10^{-08}	1.05 [1.03-1.08]	BC	[3]
rs6828523	4q34.1	174925275	A/C	C	A	*ADAM29*	intron variant	0.870	4×10^{-16}	1.11 [1.09-1.15]	BC	[3]
rs10069690	5p15.33	1279675	C/T	T	T	*TERT*	intron variant	0.260	1×10^{-10}	1.18 [1.13-1.25]	BC	[14]
								0.321	5×10^{-12}	1.15 [1.11-1.20]	BC	[2]
								0.260	7×10^{-09}	1.06 [1.04-1.09]	BC	[3]
rs7725218	5p15.33	1282299	G/A	G	A	*TERT*	intron variant	–	2×10^{-10}	–	Cancer (pleiotropy)	[1]
rs2736108*	5p15.33	1297373	T/C	C	T	*TERT - MIR4457*	upstream gene variant	0.710	2×10^{-09}	1.06 [1.04-1.09]	BC	[6]
rs13162653*	5p15.1	16187419	A/T/G	G	T	*LOC401176 - NACAP6*	downstream gene variant	0.550	1×10^{-10}	1.05 [1.03-1.08]	BC	[6]
rs2012709*	5p13.3	32567626	C/T	C	T	*SUB1*	intron variant	0.460	6×10^{-09}	1.05 [1.03-1.08]	BC	[6]
rs4415084	5p12	44662413	C/T	T	C	*LOC102723839 - RN7SL383P*	intergenic variant	0.420	8×10^{-11}	1.17 [1.11-1.22]	BC	[11]
rs10941679	5p12	44706396	A/G	A	G	*LOC102723839 - RN7SL383P*	intergenic variant	0.250	2×10^{-37}	1.13 [1.10-1.15]	BC	[3]
rs7726159*†	5p15.33	1282204	C/A	C	A	TERT	intron variant	0.340	3×10^{-08}	1.07 [1.02-1.11]	BC	[6,15]
rs16886034	5q11.2	56688029	T/G/C	T	C	*LOC101928448 - LOC105378979*	regulatory region variant	0.080	2×10^{-09}	1.36 [1.23-1.51]	BC (early onset)	[13]
rs16886113	5q11.2	56699208	T/G	T	G	*LOC101928448 - LOC105378979*	intergenic variant	0.080	4×10^{-11}	1.35 [1.23-1.47]	BC (early onset)	[13]

rs16886181	5q11.2	56733416	T/C	C	C	*LOC101928448 - LOC105378979*	intergenic variant	0.180	9x10^{-14}	1.26 [1.18-1.34]	BC (early onset)	[13]
rs889312	5q11.2	56736057	A/C	A	C	*LOC101928448 - LOC105378979*	regulatory region variant	0.290	1x10^{-08}	1.29 [–]	BC (early onset)	[13]
								0.280	7x10^{-20}	1.13 [1.10-1.16]	BC	[16]
								0.280	5x10^{-09}	1.22 [1.14-1.30]	BC	[12]
								0.280	3x10^{-36}	1.12 [1.10-1.15]	BC	[3]
rs1862626	5q11.2	56737113	G/T	T	G	*LOC101928448 - LOC105378979*	regulatory region variant	–	4x10^{-12}	–	Cancer (pleiotropy)	[1]
rs16886364	5q11.2	56826517	A/G	A	G	*MAP3K1*	intron variant	0.070	5x10^{-12}	1.36 [1.25-1.48]	BC (early onset)	[13]
rs16886397	5q11.2	56838449	A/G	A	G	*MAP3K1*	intron variant	0.070	4x10^{-12}	1.36 [1.25-1.49]	BC (early onset)	[13]
rs1017226	5q11.2	56857565	T/C	T	C	*MAP3K1*	intron variant	0.080	6x10^{-11}	1.33 [1.22-1.45]	BC (early onset)	[13]
rs2229882	5q11.2	56872885	C/T	C	T	*MAP3K1*	synonymous variant	0.060	1x10^{-14}	1.45 [1.32-1.60]	BC (early onset)	[13]
rs16886448	5q11.2	56874986	C/G	C	G	*MAP3K1*	intron variant	0.070	2x10^{-12}	1.37 [1.25-1.49]	BC (early onset)	[13]
rs3822625	5q11.2	56882284	A/G	A	G	*MAP3K1*	synonymous variant	0.070	5x10^{-12}	1.36 [1.24-1.48]	BC (early onset)	[13]
rs12655019	5q11.2	56899963	A/G	A	G	*LOC105378980*	downstream gene variant	0.100	3x10^{-10}	1.27 [1.18-1.37]	BC (early onset)	[13]
rs7726354	5q11.2	56960656	C/T	C	T	*MIER3 - LOC100130001*	intron variant	0.060	7x10^{-11}	1.37 [1.24-1.50]	BC (early onset)	[13]
rs10472076	5q11.2	58888234	T/A/C	T	C	*RAB3C - PDE4D*	intergenic variant	0.380	3x10^{-08}	1.05 [1.03-1.07]	BC	[3]
rs1353747	5q11.2	59041654	G/T	T	G	*PDE4D*	intron variant	0.905	3x10^{-08}	1.09 [1.05-1.12]	BC	[3]
rs7707921*	5q14.2	82242227	T/A	A	T	*ATG10*	intron variant	0.770	5x10^{-11}	1.08 [1.05-1.10]	BC	[6]
rs10474352	5q14.3	91436408	T/C	C	T	*ARRDC3-AS1 - RAB5CP2*	intron variant	0.482	2x10^{-09}	1.09 [1.06-1.12]	BC	[7]
rs1432679	5q33.3	158817075	T/C	C	T	*EBF1*	intron variant	0.430	2x10^{-14}	1.07 [1.05-1.09]	BC	[3]
rs11242675	6p25.3	1318643	C/T	C	C	*FOXQ1 - LINC01394*	downstream gene variant	0.610	7x10^{-09}	1.06 [1.04-1.09]	BC	[3]
rs204247	6p23	13722291	A/G	A	G	*RANBP9 - MCUR1*	intergenic variant	0.430	8x10^{-09}	1.05 [1.03-1.07]	BC	[3]
rs9257408*	6p22.1	28958443	G/C	G	G	*TRM-CAT3-2 - KRT18P1*	regulatory region variant	0.380	5x10^{-08}	1.05 [1.03-1.08]	BC	[6]
rs17529111*	6q14.1	81418669	T/C	T	C	*LOC648934 - LOC105377871*	intergenic variant	0.220	2x10^{-10}	1.06 [1.04-1.08]	BC	[6]
rs17530068	6q14.1	81483392	T/C	T	C	*LOC105377871*	intergenic variant	0.220	8x10^{-09}	1.05 [1.03-1.08]	BC	[3]
rs2180341	6q22.33	127279485	A/G	A	G	*RNF146*	intron variant	0.210	3x10^{-08}	1.41 [1.25-1.59]	BC	[17]
rs9485372	6q25.1	149287738	A/G	G	A	*TAB2*	intron variant	0.550	4x10^{-12}	1.11 [1.09-1.15]	BC	[18]
rs3757318	6q25.1	151592978	G/A	G	A	*CCDC170*	intron variant	0.070	2x10^{-21}	1.16 [1.12-1.21]	BC	[3]
rs12662670*	6q25.1	151597721	T/C/G	T	G	*CCDC170*	intron variant	0.070	7x10^{-27}	1.17 [1.13-1.22]	BC	[6]
rs2046210	6q25.1	151627231	G/A	A	A	*CCDC170 - ESR1*	intergenic variant	0.350	7x10^{-15}	1.22 [1.16-1.29]	BC	[4]
								0.423	5x10^{-16}	1.15 [1.11-1.19]	BC	[2]
								0.370	2x10^{-15}	1.29 [1.21-1.37]	BC	[19]
			A/G					0.080	5x10^{-09}	1.28 [1.18-1.39]	BC in BRCA1 mutation carriers	[20]
rs140068132	6q25.1	151633699	G/A	A	G	*CCDC170 - ESR1*	intergenic variant	0.930	9x10^{-18}	1.67 [1.49-1.89]	BC	[21]
rs9383938	6q25.1	151666222	G/T	G	T	*ESR1*	intron variant	–	2x10^{-10}	1.28 [–]	BC	[22]
rs6964587*†	7q21.2	92001306	G/T	T	T	AKAP9	missense variant	0.390	9x10^{-11}	1.03 [1.02-1.05]	BC	[6,23]
rs4593472*	7q32.3	130982362	T/C	C	T	*LINC-PINT*	intron variant	0.650	2x10^{-09}	1.05 [1.03-1.06]	BC	[6]
rs720475	7q35	144377836	A/G	G	A	*ARHGEF5*	intron variant	0.750	7x10^{-11}	1.06 [1.04-1.09]	BC	[3]
rs9693444	8p12	29652100	C/A	C	A	*RPL17P33 - LINC00589*	intergenic variant	0.320	9x10^{-14}	1.07 [1.05-1.09]	BC	[3]
rs13365225*	8p11.23	37000965	G/A	A	G	*RPL26P25 - LOC105379377*	intergenic variant	0.830	1x10^{-08}	1.05 [1.02-1.08]	BC	[6]
rs6472903	8q21.13	75318066	G/T	T	G	*HIGD1AP6 - PKMP4*	intron variant	0.820	2x10^{-17}	1.10 [1.08-1.12]	BC	[3]
rs2943559	8q21.13	75505702	A/G	A	G	*HNF4G*	intron variant	0.070	6x10^{-15}	1.13 [1.09-1.17]	BC	[3]
rs13267382*	8q23.3	116197325	G/A	A	G	*LINC00536*	intron variant	0.360	2x10^{-08}	1.05 [1.03-1.07]	BC	[6]
rs13281615	8q24.21	127343372	A/G	A	G	*CASC21, CASC8*	intron variant	0.400	5x10^{-12}	1.08 [1.05-1.11]	BC	[16]
								0.410	1x10^{-27}	1.09 [1.07-1.12]	BC	[3]
rs1562430	8q24.21	127375606	C/T	T	C	*CASC21, CASC8*	intron variant	0.600	3x10^{-11}	1.16 [1.11-1.22]	BC	[11]
rs2392780	8q24.21	127375779	G/A	G	G	*CASC21, CASC8*	intron variant	0.600	1x10^{-08}	1.15 [1.10-1.20]	BC (early onset)	[13]
rs11780156	8q24.21	128182395	C/T	C	T	*MIR1208 - RN7SKP226*	regulatory region variant	0.160	3x10^{-11}	1.07 [1.04-1.10]	BC	[3]
rs1011970	9p21.3	22062135	G/T	G	T	*CDKN2B-AS1*	intron variant	0.170	3x10^{-08}	1.09 [1.04-1.14]	BC	[12]
rs10759243	9q31.2	107543834	C/T/A	A	A	*LOC105376206 - LOC105376205*	upstream gene variant	0.390	1x10^{-08}	1.06 [1.03-1.08]	BC	[3]
rs865686	9q31.2	108126198	G/A/T	T	G	*LOC105376214*	intergenic variant	0.610	2x10^{-10}	1.12 [1.09-1.18]	BC	[11]

								0.620	$1x10^{-34}$	1.12 [1.10-1.14]	BC	[3]
rs7072776	10p12.31	21744013	G/A	A	A	*LOC107984214*	downstream gene variant	0.290	$4x10^{-14}$	1.07 [1.05-1.09]	BC	[3]
rs11814448	10p12.31	22026914	A/C	G	C	*DNAJC1 - ADIPOR1P1*	intergenic variant	0.020	$9x10^{-16}$	1.26 [1.18-1.35]	BC	[3]
rs10822013	10q21.2	62492218	C/T	C	T	*ZNF365*	intron variant	0.470	$6x10^{-09}$	1.12 [1.06-1.18]	BC	[24]
rs10995190	10q21.2	62518923	A/G	G	A	*ZNF365*	intron variant	0.850	$5x10^{-15}$	1.16 [1.10-1.22]	BC	[12]
								0.840	$1x10^{-36}$	1.16 [1.14-1.19]	BC	[3]
rs704010	10q22.3	79081391	C/T	C	T	*ZMIZ1*	intron variant	0.390	$4x10^{-09}$	1.07 [1.03-1.11]	BC	[12]
								0.380	$7x10^{-22}$	1.08 [1.06-1.10]	BC	[3]
rs7904519	10q25.2	113014168	A/G	G	G	*TCF7L2*	intron variant	0.460	$3x10^{-08}$	1.06 [1.04-1.08]	BC	[3]
rs11199914	10q26.12	121334387	T/C	T	T	*LOC105378523 - RN7SKP167*	intergenic variant	0.680	$2x10^{-08}$	1.05 [1.03-1.08]	BC	[3]
rs11200014	10q26.13	121575416	G/T/A	G	A	*FGFR2*	intron variant	–	$8x10^{-35}$	–	Cancer (pleiotropy)	[1]
rs2981579	10q26.13	121577821	G/A	G	A	*FGFR2*	intron variant	0.420	$4x10^{-31}$	1.43 [1.35-1.53]	BC	[12]
								0.400	$2x10^{-170}$	1.27 [1.24-1.29]	BC	[3]
								0.550	$3x10^{-11}$	1.19 [1.11-1.23]	BC	[4]
								0.410	$2x10^{-10}$	1.17 [1.07-1.27] (Het)	BC	[5]
rs2981578	10q26.13	121580797	A/T/C	C	T	*FGFR2*	intron variant	0.514	$1x10^{-12}$	1.23 [1.16-1.30]	BC	[25]
rs2981575	10q26.13	121586602	G/A	G	G	*FGFR2*	intron variant	0.420	$1x10^{-08}$	1.28 [1.18-1.39]	BC in BRCA2 mutation carriers	[26]
rs1219648	10q26.13	121586676	A/G	A	G	*FGFR2*	intron variant	0.420	$2x10^{-13}$	1.32 [1.22-1.42]	BC	[27]
								0.390	$6x10^{-09}$	1.17 [1.11-1.23]	BC	[4]
								0.400	1x10-^{10}	1.20 [1.07-1.42]	BC	[28]
			A/G					0.420	$1x10^{-30}$	1.31 [1.25-1.37]	BC	[11]
rs2912774	10q26.13	121589148	A/G/T	T	T	*FGFR2*	intron variant	0.440	$3x10^{-27}$	1.29 [1.23-1.35]	BC (early onset)	[13]
rs2981582	10q26.13	121592803	G/A	G	A	*FGFR2*	intron variant	0.380	$2x10^{-76}$	1.26 [1.23-1.30]	BC	[16]
								0.680	$2x10^{-08}$	1.18 [1.11-1.23]	BC	[4]
rs3817198	11p15.5	1887776	T/C	T	C	*LSP1*	intron variant	0.300	$3x10^{-09}$	1.07 [1.04-1.11]	BC	[16]
								0.310	$2x10^{-11}$	1.07 [1.05-1.09]	BC	[3]
rs3903072	11q13.1	65815595	T/G	G	T	*OVOL1 - SNX32*	regulatory region variant	0.530	$9x10^{-12}$	1.05 [1.04-1.08]	BC	[3]
rs7931342	11q13.3	69227030	T/G	G	T	*LOC105369366 - LOC105369367*	intergenic variant	–	$4x10^{-14}$	–	Cancer (pleiotropy)	[1]
rs537626	11q13.3	69492927	G/C	G	C	*LINC01488*	intergenic variant	0.180	$2x10^{-15}$	1.29 [1.21-1.37]	BC (early onset)	[13]
rs614367	11q13.3	69513996	C/T	C		*LINC01488 - CCND1*	intergenic variant	0.150	$2x10^{-63}$	1.21 [1.18-1.24]	BC	[3]
								0.150	$3x10^{-15}$	1.15 [1.10-1.20]	BC	[12]
								0.160	$1x10^{-08}$	1.34 [–]	BC (early onset)	[13]
rs78540526*	11q13.3	69516650	C/T	C	T	*LINC01488 - CCND1*	regulatory region variant	0.080	$2x10^{-86}$	1.34 [1.29-1.38]	BC	[6]
rs554219*	11q13.3	69516874	C/A/T/G	C		*LINC01488 - CCND1*	regulatory region variant	0.120	$2x10^{-81}$	1.26 [1.23-1.30]	BC	[6]
rs75915166*	11q13.3	69564393	C/A	C	A	*LINC01488 - CCND1*	intergenic variant	0.060	$1x10^{-57}$	1.31 [1.26-1.36]	BC	[6]
rs148883465	11q22.3	103813371	A/G	A	G	*LOC105369463 - LOC102723862*	intron variant	–	$3x10^{-08}$	–	Cancer (pleiotropy)	[1]
rs11820646	11q24.3	129591276	T/C	T	T	*RPS27P20 - LINC01395*	upstream gene variant	0.590	$1x10^{-09}$	1.05 [1.03-1.08]	BC	[3]
rs12422552	12p13.1	14260997	G/C	C	C	*GNAI2P1 - RPL30P11*	regulatory region variant	0.260	$4x10^{-08}$	1.05 [1.03-1.07]	BC	[3]
rs10771399	12p11.22	28002147	A/G	A	G	*PTHLH - LOC105369710*	intergenic variant		$2x10^{-08}$	1.39 [1.25-1.56]	BC (ER-, PR, and HER-)	[29]
			G/A					0.895	$2x10^{-12}$	1.20 [1.15-1.27]	BC	[2]
								0.880	$8x10^{-31}$	1.16 [1.14-1.20]	BC	[3]
rs73110464	12q13.13	52918828	C/T	C	T	*KRT8*	intron variant	–	$2x10^{-15}$	–	Cancer (pleiotropy)	[1]
rs17356907	12q22	95633983	G/A	A	G	*USP44 - PGAM1P5*	intron variant	0.700	$2x10^{-22}$	1.10 [1.08-1.12]	BC	[3]
rs1292011	12q24.21	115398717	G/A	G	G	*LOC105370003*	regulatory region variant	0.580	$9x10^{-22}$	1.09 [1.06-1.11]	BC	[3]
rs56084662	13q13.1	32295727	G/A	G	A	*FRY*	3' UTR variant	–	$4x10^{-09}$	–	Cancer (pleiotropy)	[1]
rs11571818	13q13.1	32394673	T/C	T	C	*BRCA2*	intron variant	–	$5x10^{-10}$	–	Cancer (pleiotropy)	[1]
rs11571833	13q13.1	32398489	A/T	A	T	*BRCA2*	stop gained	0.008	$5x10^{-08}$	1.26 [1.14-1.39]	BC	[3]
								–	$8x10^{-12}$	1.6 [–]	Cancer	[1]
rs2236007	14q13.3	36663564	A/G	G	A	*PAX9*	intron variant	0.790	$2x10^{-13}$	1.08 [1.05-1.10]	BC	[3]
rs2588809	14q24.1	68193711	C/T	T	T	*RAD51B*	intron variant	0.160	$1x10^{-10}$	1.08 [1.05-1.11]	BC	[3]

rs1314913	14q24.1	68232877	C/T	C	T	*RAD51B*	intron variant	–	3x10^{-13}	1.57 [1.39-1.77]	BC (male)	[30]
rs11844632	14q24.1	68559662	G/A	A	A	*RAD51B*	intron variant	–	3x10^{-10}	–	Cancer (pleiotropy)	[1]
rs999737	14q24.1	68567965	T/C	C	T	*RAD51B*	intron variant	0.770	3x10^{-19}	1.09 [1.06-1.11]	BC	[3]
rs941764	14q32.11	91374725	A/G	G	G	*CCDC88C*	intron variant	0.340	4x10^{-10}	1.06 [1.04-1.09]	BC	[3]
rs11627032*	14q32.12	92637727	C/T	T	C	*RIN3*	intron variant	0.740	4x10^{-09}	1.06 [1.04-1.09]	BC	[6]
rs2290203	15q26.1	90968837	A/G	A	A	*PRC1, PRC1-AS1*	intron variant	0.504	4x10^{-08}	1.08 [1.05-1.11]	BC	[7]
rs4784223	16q12.1	52541995	A/G	A	G	*TOX3*	intron variant	0.290	6x10^{-21}	1.27 [1.21-1.34]	BC (early onset)	[13]
rs3803662	16q12.1	52552429	G/A	G	A	*CASC16*	non-coding transcript exon variant	0.522	3x10^{-11}	1.21 [1.15-1.28]	BC	[25]
								0.260	2x10^{-114}	1.24 [1.21-1.27]	BC	[3]
								0.270	1x10^{-09}	1.16 [1.07-1.27] (Het)	BC	[25]
								0.270	6x10^{-19}	1.28 [1.21-1.35]	BC	[9]
								0.250	1x10^{-36}	1.20 [1.16-1.24]	BC	[16]
								0.267	6x10^{-13}	1.14 [1.10-1.18]	BC	[2]
								0.260	3x10^{-15}	1.30 [1.22-1.39]	BC	[12]
								–	4x10^{-15}	1.50 [1.35-1.66]	BC (male)	[30]
rs4784227	16q12.1	52565276	C/T	C	T	*CASC16*	intron variant	0.310	3x10^{-09}	1.38 [1.24-1.54]	BC	[21]
								0.240	1x10^{-28}	1.24 [1.20-1.29]	BC	[31]
rs12922061	16q12.2	52601088	C/T	C	T	*CASC16*	intron variant	0.241	4x10^{-10}	1.23 [1.15-1.31]	BC	[25]
rs3112612	16q12.2	52601252	G/A	A	G	*CASC16*	intron variant	0.430	4x10^{-10}	1.15 [1.10-1.21]	BC	[11]
rs17817449	16q12.2	53779455	G/T	G	G	*FTO*	intron variant	0.600	6x10^{-14}	1.08 [1.05-1.10]	BC	[3]
rs11075995	16q12.2	53821379	T/A	T	A	*FTO*	intron variant	0.240	4x10^{-08}	1.11 [1.07-1.15]	BC	[2]
rs13329835	16q23.2	80616908	A/G	G	G	*CDYL2*	intron variant	0.220	2x10^{-16}	1.08 [1.05-1.10]	BC	[3]
rs146699004*	17q11.2	29230520	GGT/G	–	–	No mapped genes		0.80	3x10^{-8}	1.08 [1.04-1.10]	BC	[6]
rs12601991	17q12	37741642	G/T	G	T	*HNF1B*	intron variant	–	1x10^{-09}	1.10 [–]	Cancer	[1]
								–	7x10^{-28}	–	Cancer (pleiotropy)	[1]
rs6504950	17q22	54979110	A/G	A	A	*STXBP4*	intron variant	0.720	2x10^{-13}	1.06 [1.04-1.09]	BC	[3]
rs745570*	17q25.3	79807926	G/A	G	A	*CBX8 - LOC102723961*	intergenic variant	0.500	1x10^{-09}	1.05 [1.03-1.08]	BC	[6]
rs527616	18q11.2	26757460	C/G	G	C	*PCAT18 - LOC105372035*	intron variant	0.620	2x10^{-10}	1.05 [1.03-1.08]	BC	[3]
rs1436904	18q11.2	26990703	G/T	T	G	*CHST9*	intron variant	0.600	3x10^{-08}	1.04 [1.02-1.06]	BC	[3]
rs6507583*	18q12.3	44819625	G/A	G	G	*SETBP1*	intron variant	0.930	3x10^{-08}	1.10 [1.05-1.14]	BC	[6]
rs8170	19p13.11	17278895	G/A	G	A	*BABAM1*	synonymous variant	0.170	2x10^{-09}	1.26 [1.17-1.35]	BC	[32]
								0.191	9x10^{-13}	1.15 [1.11-1.20]	BC	[2]
			A/G					0.480	4x10^{-13}	1.19 [1.14-1.25]	BC in BRCA1 mutation carriers	[20]
rs4808075	19p13.11	17279482	T/C	C	C	*BABAM1 - ANKLE1*	intron variant	–	2x10^{-09}	–	Cancer (pleiotropy)	[1]
rs8100241	19p13.11	17282085	A/G	G	A	*ANKLE1*	missense variant	–	4x10^{-08}	1.14 [–]	BC	[22]
rs2363956	19p13.11	17283315	T/G	T	G	*ANKLE1*	missense variant		2x10^{-08}	1.22 [1.14-1.30]	BC (ER-, PR, and HER-)	[29]
rs4808801	19p13.11	18460331	G/A	A	G	*ELL*	intron variant	0.650	5x10^{-15}	1.08 [1.05-1.10]	BC	[3]
rs3760982	19q13.31	43782361	G/A	G	A	*KCNN4 - LOC107987268*	upstream gene variant	0.460	2x10^{-10}	1.06 [1.04-1.08]	BC	[3]
rs2284378	20q11.22	34000289	C/T	C	T	*RALY*	intron variant	0.310	1x10^{-08}	1.16 [1.10-1.22]	BC	[22]
rs2300206	20q11.22	34002002	C/G	C	G	*RALY*	intron variant	–	2x10^{-08}	1.11 [–]	Cancer	[1]
rs11907546	20q11.22	34131991	A/T/C	C	C	*RPS2P1 - ASIP*	regulatory region variant	–	3x10^{-09}	1.11 [–]	Cancer	[1]
rs2823093	21q21.1	15148511	A/G	A	A	*LOC107985483 - LOC105372739*	intergenic variant	0.730	7x10^{-16}	1.09 [1.06-1.11]	BC	[3]
rs16992204	21q22.12	34738904	T/C	C	C	*LOC107985515*	upstream gene variant	0.120	5x10^{-08}	1.13 [1.07-1.18]	BC	[4]
rs17879961*†	22q12.1	28725099	A/C/G	A	G	*CHEK2*	missense variant	0.005	1x10^{-8}	1.26 [1.11-1.42]	BC	[6,23]
rs132390	22q12.2	29225488	T/C	T	C	*EMID1*	intron variant	0.036	3x10^{-09}	1.12 [1.07-1.18]	BC	[3]
rs6001930	22q13.1	40480230	T/C	C	C	*MKL1*	intron variant	0.110	9x10^{-19}	1.12 [1.09-1.16]	BC	[3]

* Retrieved from Michailidou et al., 2015
† SNP did not reach genome-wide significance threshold at Michailidou et al., 2015 but was later associated with risk (reported in table; $P \leq 5x10^{-8}$)
[1] dbSNP Build 150
[2] GRCh38
[3] Protective/risk allele, based on the forward strand (Alleles were retrieved from the Ensembl database v91; risk allele is as reported at the GWAS Catalog)
[4] Ancestral allele, retrieved from the Ensembl database v92
[5] Minor allele, retrieved from the Ensembl database v92
[6] Risk allele frequency, reported at the GWAS Catalog
[7] Odds Ratio [95% Confidence Interval], reported at the GWAS Catalog
[8] Trait reported at the GWAS Catalog

Table S2. Breast cancer risk associated SNPs located near miRNA genes.

Table S2a. Proxy SNPs ($r^2 \geq 0.8$) located downstream or upstream miRNA genes.

SNP	Locus	Position[1]	Alleles[2]	AA[3]	MA[4]	Gene	Context	GWAS SNP	Distance	r^2	D'
rs2004659	1q21.1	145846884	A/G	A	G	*MIR6736*	downstream gene variant	rs12405132*	56800	0.962	1.000
rs9286836	1q21.1	145846532	A/C/G	G	G	*MIR6736*	downstream gene variant	rs12405132*	56448	0.926	1.000
rs56041970	2q34	212423018	T/C	T	C	*MIR548F2*	downstream gene variant	rs13393577	9121	1.000	1.000
rs13413335	2q34	212423940	A/C	A	C	*MIR548F2*	downstream gene variant	rs13393577	8199	0.850	1.000
rs10197215	2q34	212425362	A/G	A	G	*MIR548F2*	downstream gene variant	rs13393577	6777	1.000	1.000
rs10166403	2q34	212426475	A/G	A	G	*MIR548F2*	upstream gene variant	rs13393577	5664	0.810	1.000
rs10202760	2q34	212426630	T/C	T	C	*MIR548F2*	upstream gene variant	rs13393577	5509	1.000	1.000
rs77257332	2q34	212430962	A/G	A	G	*MIR548F2*	upstream gene variant	rs13393577	1177	1.000	1.000
rs34344935	7q21.2	92206122	G/A	G	A	*MIR1285-1*	upstream gene variant	rs6964587*†	204816	0.967	1.000
rs1121948	8q24.31	128152810	A/G	A	G	*MIR1208*	downstream gene variant	rs11780156	29585	0.843	1.000
rs72843959	11p15.5	1885061	C/G	C	G	*MIR7847*	downstream gene variant	rs3817198	2715	0.964	1.000
rs4911146	20q11.32	34052241	C/G	G	C	*MIR4755*	downstream gene variant	rs2284378	51952	1.000	1.000
rs3787230	20q11.32	34052466	A/G	A	A	*MIR4755*	downstream gene variant	rs2284378	52177	0.961	1.000

Table S2b. Proxy SNPs ($0.2 \leq r^2 < 0.8$) located downstream or upstream miRNA genes.

SNP	Locus	Position[1]	Alleles[2]	AA[3]	MA[4]	Gene	Context	GWAS SNP	Distance	r^2	D'
rs35766535	1p36.22	10224601	T/C	T	C	*MIR1273D*	upstream gene variant	rs616488	281556	0.244	0.502
rs61775887	1p36.22	10224675	T/C	C	C	*MIR1273D*	upstream gene variant	rs616488	281482	0.285	0.573
rs12122468	1p36.22	10228364	A/T	T	T	*MIR1273D*	downstream gene variant	rs616488	277793	0.285	0.573
rs55676616	1p36.22	10230068	T/C	C	C	*MIR1273D*	downstream gene variant	rs616488	276089	0.285	0.573
rs17396382	1p36.22	10230210	T/C	T	C	*MIR1273D*	downstream gene variant	rs616488	275947	0.244	0.502
rs17400510	1p36.22	10230363	T/C	T	C	*MIR1273D*	downstream gene variant	rs616488	275794	0.261	0.530
rs12129035	1p36.22	10231108	G/A	G	A	*MIR1273D*	downstream gene variant	rs616488	275049	0.244	0.502
rs12751375	1p36.22	10231816	C/G	C	G	*MIR1273D*	downstream gene variant	rs616488	274341	0.271	0.521
rs11133727	5p15.33	1306650	C/G	C	–	*MIR4457*	downstream gene variant	rs2736108*	9277	0.246	0.682
rs61574973	5p15.33	1309053	T/C	C	T	*MIR4457*	downstream gene variant	rs2736108*	11680	0.225	0.700
rs6554758	5p15.33	1310037	G/A	G	G	*MIR4457*	upstream gene variant	rs2736108*	12664	0.282	0.744
rs6866294	5p15.33	1311578	T/C	C	T	*MIR4457*	upstream gene variant	rs2736108*	14205	0.264	0.695
rs13356727	5p15.33	1312342	G/A	G	G	*MIR4457*	upstream gene variant	rs2736108*	14969	0.259	0.689
rs759649	8q24.21	128146998	G/A	A	G	*MIR1208*	upstream gene variant	rs11780156	35397	0.214	1.000
rs7814495	8q24.21	128149207	G/C	G	C	*MIR1208*	upstream gene variant	rs11780156	33188	0.660	1.000
rs10956412	8q24.21	128150251	A/C/G	A	C	*MIR1208*	downstream gene variant	rs11780156	32144	0.598	0.851
rs12676304	8q24.21	128151049	A/C	A	C	*MIR1208*	downstream gene variant	rs11780156	31346	0.550	1.000
rs759651	8q24.21	128152042	T/A/C	T	C	*MIR1208*	downstream gene variant	rs11780156	30353	0.550	1.000
rs1121946	8q24.21	128152952	T/G	T	G	*MIR1208*	downstream gene variant	rs11780156	29443	0.714	1.000
rs12675643	8q24.21	128153446	T/A	T	A	*MIR1208*	downstream gene variant	rs11780156	28949	0.550	1.000
rs10956413	8q24.21	128154939	C/A	C	A	*MIR1208*	downstream gene variant	rs11780156	27456	0.527	1.000
rs5011832	10p12.31	21493913	T/C	C	C	*MIR1915*	downstream gene variant	rs7072776	250100	0.623	0.905
rs12770228	10p12.31	21494705	G/A	G	A	*MIR1915*	downstream gene variant	rs7072776	249308	0.697	0.869
rs35106872	10p12.31	21494823	A/G	G	G	*MIR1915*	downstream gene variant	rs7072776	249190	0.613	0.853
rs12357321	10p12.31	21501547	G/A	G	A	*MIR1915*	upstream gene variant	rs7072776	242466	0.687	0.864
rs10839819	11p15.5	1854956	C/T	C	T	*MIR4298*	downstream gene variant	rs3817198	32820	0.227	0.900
rs11041481	11p15.5	1855324	A/G	G	G	*MIR4298*	downstream gene variant	rs3817198	32452	0.227	0.900
rs10839821	11p15.5	1855365	G/A	A	A	*MIR4298*	downstream gene variant	rs3817198	32411	0.227	0.900
rs7934551	11p15.5	1855487	C/T	C	T	*MIR4298*	downstream gene variant	rs3817198	32289	0.227	0.900
rs10769814	11p15.5	1855725	A/C	C	A	*MIR4298*	downstream gene variant	rs3817198	32051	0.272	0.574

rs7112859	11p15.5	1855841	C/T	C	T	*MIR4298*	downstream gene variant	rs3817198	31935	0.227	0.900
rs869227	11p15.5	1856208	A/G	G	G	*MIR4298*	downstream gene variant	rs3817198	31568	0.227	0.900
rs2001487	11p15.5	1856282	C/G	G	G	*MIR4298*	downstream gene variant	rs3817198	31494	0.227	0.900
rs907614	11p15.5	1856303	T/C	T	T	*MIR4298*	downstream gene variant	rs3817198	31473	0.272	0.574
rs603073	11p15.5	1856617	T/C	C	C	*MIR4298*	downstream gene variant	rs3817198	31159	0.216	0.896
rs2685284	11p15.5	1856843	G/A	G	A	*MIR4298*	downstream gene variant	rs3817198	30933	0.227	0.900
rs72843933	11p15.5	1857176	C/T	C	T	*MIR4298*	downstream gene variant	rs3817198	30600	0.220	0.561
rs1717769	11p15.5	1857232	T/C	C	C	*MIR4298*	downstream gene variant	rs3817198	30544	0.227	0.900
rs474016	11p15.5	1857344	A/G	G	G	*MIR4298*	downstream gene variant	rs3817198	30432	0.227	0.900
rs599774	11p15.5	1857406	A/G	G	G	*MIR4298*	downstream gene variant	rs3817198	30370	0.227	0.900
rs567602	11p15.5	1859246	T/C	C	T	*MIR4298*	downstream gene variant	rs3817198	28530	0.231	0.509
rs571122	11p15.5	1859581	G/A	A	G	*MIR4298*	upstream gene variant	rs3817198	28195	0.249	0.538
rs587074	11p15.5	1859841	G/A	G	G	*MIR4298*	upstream gene variant	rs3817198	27935	0.206	0.471
rs587961	11p15.5	1860026	T/C	C	T	*MIR4298*	upstream gene variant	rs3817198	27750	0.201	0.503
rs2048540	11p15.5	1861018	T/C	T	T	*MIR4298*	upstream gene variant	rs3817198	26758	0.249	0.538
rs4980392	11p15.5	1862625	T/C	T	T	*MIR4298*	upstream gene variant	rs3817198	25151	0.272	0.574
rs72843938	11p15.5	1863123	G/A	G	A	*MIR4298*	upstream gene variant	rs3817198	24653	0.372	0.856
rs4980383	11p15.5	1880867	C/T	C	T	*MIR7847*	downstream gene variant	rs3817198	6909	0.385	0.876
rs620315	11p15.5	1881245	G/A	G	A	*MIR7847*	downstream gene variant	rs3817198	6531	0.208	0.894
rs621679	11p15.5	1902768	G/A	G	A	*MIR7847*	downstream gene variant	rs3817198	6238	0.208	0.894
rs61868798	11p15.5	1883201	G/A	G	A	*MIR7847*	downstream gene variant	rs3817198	4575	0.231	0.529
rs661348	11p15.5	1884062	T/C	T	C	*MIR7847*	downstream gene variant	rs3817198	3714	0.294	0.922
rs3817197	11p15.5	1884944	G/A	A	G	*MIR7847*	downstream gene variant	rs3817198	2832	0.397	1.000
rs2009453	11q13.1	65632057	C/T	C	T	*MIR4690*	upstream gene variant	rs3903072	183538	0.299	0.680
rs6591183	11q13.1	65633417	A/G	G	G	*MIR4690*	upstream gene variant	rs3903072	182178	0.264	0.629
rs10896026	11q13.1	65633948	C/T	C	T	*MIR4690*	upstream gene variant	rs3903072	181647	0.247	0.562
rs931127	11q13.1	65637829	G/A	G	A	*MIR4690*	downstream gene variant	rs3903072	177766	0.299	0.680
rs2306362	11q13.1	65638039	G/A/T	G	T	*MIR4690*	downstream gene variant	rs3903072	177556	0.247	0.562
rs2306364	11q13.1	65644996	G/A/C/T	G	A	*MIR4489*	upstream gene variant	rs3903072	170599	0.350	0.602
rs746429	11q13.1	65649963	G/A	G	A	*MIR4489*	downstream gene variant	rs3903072	165632	0.247	0.562
rs1466462	11q13.1	65651893	G/C	G	C	*MIR4489*	downstream gene variant	rs3903072	163702	0.226	0.510
rs67649296	18q11.2	26591463	C/A	C	A	*MIR8057*	downstream gene variant	rs527616	165997	0.215	0.559
rs6059856	20q11.22	34470149	G/C	C	C	*MIR644A*	downstream gene variant	rs2284378	469859	0.413	0.933
rs6087587	20q11.22	34470823	G/T	G	T	*MIR644A*	downstream gene variant	rs2284378	470533	0.413	0.933
rs79035401	22q13.1	40816545	T/C	T	C	*MIR4766*	upstream gene variant	rs6001930	336315	0.379	1.000

* Retrieved from Michailidou et al., 2015

† SNP did not reach genome-wide significance threshold at Michailidou et al., 2015 but was later associated with risk ($P \leq 5x10^{-8}$).

[1] GRCh38

[2] Retrieved from the Ensembl database v92

[3] Ancestral allele, retrieved from the Ensembl database v92

[4] Minor allele, retrieved from the Ensembl database v92

Table S3. Comparison of miRNA-target prediction algorithms. Existing data or predictions are indicated by a cross.

Algorithm	Version (Release Year)	Method				Search	Availability		Ref.
		Seed Complementarity	Thermodynamics	Conservation	Genomic Context		Online	Source code or Software	
**TargetScan**	7.1 (2015)	x	x	x	x	3'UTR	www.targetscan.org	x (Perl)	[33]
**miRanda**	3.3a (2010)		x			5'UTR, CDS, 3'UTR	www.microrna.org	x	[34,35]
**PicTar**	— (2007)		x	x		3'UTR	pictar.mdc-berlin.de		[36]
**DIANA microT-CDS**	5.0 (2012)	x	x	x	x	CDS, 3'UTR	www.microrna.gr/microT-CDS		[37,38]
**RNAHybrid**	— (2004)		x			3'UTR	bibiserv2.cebitec.uni-bielefeld.de/rnahybrid	x	[39,40]
**PITA**	6 (2008)	x	x	x		3'UTR	genie.weizmann.ac.il/pubs/mir07	x (Perl)	[41]
**miRTar**		x	x	x	x	5'UTR, CDS, 3'UTR	mirtar.mbc.nctu.edu.tw/human		[42]

Table S4. 3'UTR-located BC risk-associated variants. Existing data or predictions are indicated by a cross.

Locus	GWAS SNP	LD[1]		SNP	Alleles	A.A.[2]	M.A.[3]	Gene ID	Gene	Transcript ID	TargetScan v7.1	miRanda 3.3a
		r^2	D'									
1q21.1	rs12405132	0.851	0.959	rs17354678	T/C	T	C	ENSG00000265491	*RNF115*	ENST00000582693	x	x
		0.851	0.959	rs12123298	G/A/C	G	A				x	x
		0.961	1	rs17352469	T/C	T	C				x	x
		0.961	1	rs2231375	C/T	C	T	ENSG00000117281	*CD160*	ENST00000616463	x	x
		0.961	1	rs1778523	G/C	G	C				x	x
1q32.1	rs4245739	0.821	0.959	rs4245738	C/T	T	C	ENSG00000198625	*MDM4*	ENST00000367180	x	x
		1	1	rs4245739	C/A	A	C	ENSG00000198625	*MDM4*	ENST00000391947	x	x
		1	1	rs4245739	C/A	A	C	ENSG00000198625	*MDM4*	ENST00000621032	x	x
		1	1	rs4245739	C/A	A	C	ENSG00000198625	*MDM4*	ENST00000612738	x	x
		1	1	rs4245739	C/A	A	C	ENSG00000198625	*MDM4*	ENST00000616250	x	x
		1	1	rs4245739	C/A	A	C	ENSG00000198625	*MDM4*	ENST00000454264	x	x
		1	1	rs4245739	C/A	A	C	ENSG00000198625	*MDM4*	ENST00000367182	x	x
		1	1	rs4245739	C/A	A	C	ENSG00000198625	*MDM4*	ENST00000367183	x	x
		1	1	rs4245739	C/A	A	C	ENSG00000198625	*MDM4*	ENST00000614459	x	x
		0.861	1	rs10900596	T/C	C	T	ENSG00000198625	*MDM4*	ENST00000612738	x	x
		0.861	1	rs10900596	T/C	C	T	ENSG00000198625	*MDM4*	ENST00000367183	x	x
		0.861	1	rs10900596	T/C	C	T	ENSG00000198625	*MDM4*	ENST00000367182	x	x
		0.861	1	rs10900596	T/C	C	T	ENSG00000198625	*MDM4*	ENST00000391947	x	x
		0.861	1	rs10900596	T/C	C	T	ENSG00000198625	*MDM4*	ENST00000614459	x	x
		0.861	1	rs10900596	T/C	C	T	ENSG00000198625	*MDM4*	ENST00000616250	x	x
		0.861	1	rs10900596	T/C	C	T	ENSG00000198625	*MDM4*	ENST00000454264	x	x
		0.861	1	rs10900596	T/C	C	T	ENSG00000198625	*MDM4*	ENST00000621032	x	x
		0.861	1	rs10900597	C/T	C	C	ENSG00000198625	*MDM4*	ENST00000621032	x	x
		0.861	1	rs10900597	C/T	C	C	ENSG00000198625	*MDM4*	ENST00000367183	x	x
		0.861	1	rs10900597	C/T	C	C	ENSG00000198625	*MDM4*	ENST00000454264	x	x
		0.861	1	rs10900597	C/T	C	C	ENSG00000198625	*MDM4*	ENST00000614459	x	x
		0.861	1	rs10900597	C/T	C	C	ENSG00000198625	*MDM4*	ENST00000367182	x	x
		0.861	1	rs10900597	C/T	C	C	ENSG00000198625	*MDM4*	ENST00000391947	x	x
		0.861	1	rs10900597	C/T	C	C	ENSG00000198625	*MDM4*	ENST00000616250	x	x
		0.861	1	rs10900597	C/T	C	C	ENSG00000198625	*MDM4*	ENST00000612738	x	x
3p24.1	rs4973768	1	1	rs4973768	C/T	T	T	ENSG00000033867	*SLC4A7*	ENST00000428386	x	x
		1	1	rs4973768	C/T	T	T	ENSG00000033867	*SLC4A7*	ENST00000295736	x	x

		1	1	rs4973768	C/T	T	T	ENSG00000033867	*SLC4A7*	ENST00000419036	x	x
		1	1	rs4973768	C/T	T	T	ENSG00000033867	*SLC4A7*	ENST00000425128	x	x
	rs653465	0.839	0.963	rs4973768	C/T	T	T	ENSG00000033867	*SLC4A7*	ENST00000419036	x	x
		0.839	0.963	rs4973768	C/T	T	T	ENSG00000033867	*SLC4A7*	ENST00000295736	x	x
		0.839	0.963	rs4973768	C/T	T	T	ENSG00000033867	*SLC4A7*	ENST00000428386	x	x
		0.839	0.963	rs4973768	C/T	T	T	ENSG00000033867	*SLC4A7*	ENST00000425128	x	x
	rs4973768	1	1	rs1051545	T/C	C	C	ENSG00000033867	*SLC4A7*	ENST00000295736	x	x
		1	1	rs1051545	T/C	C	C	ENSG00000033867	*SLC4A7*	ENST00000425128	x	x
		1	1	rs1051545	T/C	C	C	ENSG00000033867	*SLC4A7*	ENST00000419036	x	x
		1	1	rs1051545	T/C	C	C	ENSG00000033867	*SLC4A7*	ENST00000428386	x	x
	rs653465	0.839	0.963	rs1051545	T/C	C	C	ENSG00000033867	*SLC4A7*	ENST00000295736	x	x
		0.839	0.963	rs1051545	T/C	C	C	ENSG00000033867	*SLC4A7*	ENST00000428386	x	x
		0.839	0.963	rs1051545	T/C	C	C	ENSG00000033867	*SLC4A7*	ENST00000425128	x	x
		0.839	0.963	rs1051545	T/C	C	C	ENSG00000033867	*SLC4A7*	ENST00000419036	x	x
3p14.1	rs1053338	0.895	1	rs3733126	C/T	T	T	ENSG00000285258	*ATXN7*	ENST00000295900	x	x
		0.895	1	rs3733126	C/T	T	T	ENSG00000163635	*ATXN7*	ENST00000538065	x	x
		0.837	0.941	rs1046025	C/T	T	T	ENSG00000163636	*PSMD6*	ENST00000480205	x	x
		0.837	0.941	rs1046025	C/T	T	T	ENSG00000163636	*PSMD6*	ENST00000482510	x	x
		0.837	0.941	rs1046025	C/T	T	T	ENSG00000163636	*PSMD6*	ENST00000492933	x	x
		0.837	0.941	rs1046025	C/T	T	T	ENSG00000163636	*PSMD6*	ENST00000295901	x	x
		0.837	0.941	rs1046025	C/T	T	T	ENSG00000163636	*PSMD6*	ENST00000394431	x	x
5q11.2	rs12655019	0.92	1	rs1466010	A/G	A	G	ENSG00000155542	*SETD9*	ENST00000418299	x	x
		0.92	1	rs12654125	G/A	G	A	ENSG00000155545	*MIER3*	ENST00000381226	x	x
		0.92	1	rs12654125	G/A	G	A	ENSG00000155545	*MIER3*	ENST00000452157	x	x
		0.92	1	rs12654125	G/A	G	A	ENSG00000155545	*MIER3*	ENST00000381199	x	x
		0.92	1	rs12654125	G/A	G	A	ENSG00000155545	*MIER3*	ENST00000381213	x	x
		0.92	1	rs3756586	A/G	G	G	ENSG00000155545	*MIER3*	ENST00000381226	x	x
		0.92	1	rs3756586	A/G	G	G	ENSG00000155545	*MIER3*	ENST00000381213	x	x
		0.92	1	rs3756586	A/G	G	G	ENSG00000155545	*MIER3*	ENST00000381199	x	x
		0.92	1	rs3756586	A/G	G	G	ENSG00000155545	*MIER3*	ENST00000452157	x	x
		0.92	1	rs16886496	T/C	T	C	ENSG00000155545	*MIER3*	ENST00000381226	x	x
		0.92	1	rs16886496	T/C	T	C	ENSG00000155545	*MIER3*	ENST00000381199	x	x
		0.92	1	rs16886496	T/C	T	C	ENSG00000155545	*MIER3*	ENST00000381213	x	x
		0.92	1	rs16886496	T/C	T	C	ENSG00000155545	*MIER3*	ENST00000452157	x	x
5q14.2	rs7707921	0.881	1	rs73136782	T/G	T	G	ENSG00000152348	*ATG10*	ENST00000355178	x	x
		1	1	rs6884232	G/A	A	G	ENSG00000152348	*ATG10*	ENST00000282185	x	x
		1	1	rs6884232	G/A	A	G	ENSG00000152348	*ATG10*	ENST00000458350	x	x
		1	1	rs1019806	G/A	A	G	ENSG00000152348	*ATG10*	ENST00000282185	x	x
6q22.33	rs2180341	1	1	rs9321073	C/T	T	C	ENSG00000118518	*RNF146*	ENST00000356799	x	x
		1	1	rs9321073	C/T	T	C	ENSG00000118518	*RNF146*	ENST00000309649	x	x
		1	1	rs9321073	C/T	T	C	ENSG00000118518	*RNF146*	ENST00000368314	x	x
		1	1	rs9321073	C/T	T	C	ENSG00000118518	*RNF146*	ENST00000616343	x	x
6q25.1	rs12662670	0.892	1	rs3734805	A/C	A	C	ENSG00000120262	*CCDC170*	ENST00000239374	x	x
		0.892	1	rs9383935	C/T	C	T	ENSG00000120262	*CCDC170*	ENST00000239374	x	x
		0.892	1	rs9383589	A/G	A	G	ENSG00000120262	*CCDC170*	ENST00000239374	x	x
	rs2046210	0.821	1	rs3734806	G/A	G	A	ENSG00000120262	*CCDC170*	ENST00000239374	x	x
		0.821	1	rs3757322	T/G	T	G	ENSG00000120262	*CCDC170*	ENST00000239374	x	x
7q21.2	rs6964587	1	1	rs55745934	T/C	T	C	ENSG00000127914	*AKAP9*	ENST00000619023[†]		x
		1	1	rs10225885	A/G	A	G	ENSG00000127914	*AKAP9*	ENST00000619023[†]		x
		1	1	rs10225892	A/G	G	G	ENSG00000127914	*AKAP9*	ENST00000619023[†]		x

		1	1	rs28584017	G/A	G	A	ENSG00000127914	*AKAP9*	ENST00000435423†		x
		1	1	rs28584017	G/A	G	A	ENSG00000127914	*AKAP9*	ENST00000358100†		x
		1	1	rs4265	C/T	C	T	ENSG00000127914	*AKAP9*	ENST00000358100†		x
		1	1	rs4265	C/T	C	T	ENSG00000127914	*AKAP9*	ENST00000435423†		x
11q13.1	rs3903072	0.837	0.963	rs633800	G/A	G	A	ENSG00000172638	*EFEMP2*	ENST00000530850*		x
		0.837	0.963	rs633800	G/A	G	A	ENSG00000172638	*EFEMP2*	ENST00000533347*		x
13q13.1	rs56084662	–	–	rs56084662	G/A	G	A	ENSG00000073910	*FRY*	ENST00000380250	x	x
		–	–	rs56084662	G/A	G	A	ENSG00000073910	*FRY*	ENST00000645780	x	x
		–	–	rs56084662	G/A	G	A	ENSG00000073910	*FRY*	ENST00000542859	x	x
		–	–	rs56084662	G/A	G	A	ENSG00000073910	*FRY*	ENST00000642040	x	x
		–	–	rs56084662	G/A	G	A	ENSG00000073910	*FRY*	ENST00000647500	x	X
15q26.1	rs2290203	1	1	rs2290203	G/A	A	A	ENSG00000284946	–	ENST00000643536*		x
		0.938	1	rs2301826	C/T	T	T	ENSG00000284946	–	ENST00000647331*		x
		0.938	1	rs2301826	C/T	T	T	ENSG00000284946	–	ENST00000643536*		x
17q22	rs6504950	0.83	1	rs3087650	G/A	G	A	ENSG00000166260	*COX11*	ENST00000576370*		x
		0.83	1	rs3087650	G/A	G	A	ENSG00000166260	*COX11*	ENST00000574821*		x
		0.83	1	rs3087650	G/A	G	A	ENSG00000166260	*COX11*	ENST00000572558*		x
		0.83	1	rs1802212	A/C	A	C	ENSG00000141198	*TOM1L1*	ENST00000575882	x	x
		0.83	1	rs1802212	A/C	A	C	ENSG00000141198	*TOM1L1*	ENST00000445275	x	x
		0.83	1	rs1802212	A/C	A	C	ENSG00000166260	*COX11*	ENST00000299335	x	x
		0.83	1	rs1802212	A/C	A	C	ENSG00000141198	*TOM1L1*	ENST00000348161	x	x
		0.83	1	rs1802212	A/C	A	C	ENSG00000141198	*TOM1L1*	ENST00000536554	x	x
		0.83	1	rs1802212	A/C	A	C	ENSG00000166260	*COX11*	ENST00000576370	x	x
		0.83	1	rs1802212	A/C	A	C	ENSG00000141198	*TOM1L1*	ENST00000572158	x	x
		0.83	1	rs1802212	A/C	A	C	ENSG00000141198	*TOM1L1*	ENST00000571319	x	x
		0.83	1	rs17817901	A/G	A	G	ENSG00000141198	*TOM1L1*	ENST00000445275	x	x
		0.83	1	rs17817901	A/G	A	G	ENSG00000166260	*COX11*	ENST00000576370	x	x
		0.83	1	rs17817901	A/G	A	G	ENSG00000166260	*COX11*	ENST00000299335	x	x
		0.83	1	rs17817901	A/G	A	G	ENSG00000141198	*TOM1L1*	ENST00000571319	x	x
		0.83	1	rs17817901	A/G	A	G	ENSG00000141198	*TOM1L1*	ENST00000536554	x	x
		0.83	1	rs17817901	A/G	A	G	ENSG00000141198	*TOM1L1*	ENST00000348161	x	x
		0.83	1	rs17817901	A/G	A	G	ENSG00000141198	*TOM1L1*	ENST00000575882	x	x
19p13.11	rs8170	1	1	rs8170	G/A	G	A	ENSG00000105393	*BABAM1*	ENST00000601232*		x
	rs2363956	1	1	rs8100241	G/A	G	A	ENSG00000269307	–	ENST00000596542*		x
	rs8100241	1	1	rs8100241	G/A	G	A	ENSG00000269307	–	ENST00000596542*		x
	rs2363956	1	1	rs8108174	T/A	T	A	ENSG00000269307	–	ENST00000596542*		x
		1	1	rs8108174	T/A	T	A	ENSG00000160117	*ANKLE1*	ENST00000404085*		x
	rs8100241	1	1	rs8108174	T/A	T	A	ENSG00000269307	–	ENST00000596542*		x
	rs8100241	1	1	rs8108174	T/A	T	A	ENSG00000160117	*ANKLE1*	ENST00000404085*		x
	rs2363956	1	1	rs2363956	T/G	T	G	ENSG00000160117	*ANKLE1*	ENST00000404085*		x
	rs8100241	1	1	rs2363956	T/G	T	G	ENSG00000160117	*ANKLE1*	ENST00000404085*		x
	rs8170	1	1	rs10425939	C/T	C	T	ENSG00000160117	*ANKLE1*	ENST00000404085	x	x
	rs8170	1	1	rs10425939	C/T	C	T	ENSG00000160117	*ANKLE1*	ENST00000404261	x	x
	rs4808075	1	1	rs4808616	C/A	C	A	ENSG00000127220	*ABHD8*	ENST00000247706	x	x
	rs8170	0.859	0.95	rs11540855	A/G/T	A	G	ENSG00000127220	*ABHD8*	ENST00000247706	x	x
	rs4808801	1	1	rs10405636	A/C	C	C	ENSG00000130511	*SSBP4*	ENST00000607020*		x
		1	1	rs10405636	A/C	C	C	ENSG00000130511	*SSBP4*	ENST00000601614*		x
		0.965	1	rs2385088	A/G	G	G	ENSG00000105655	*ISYNA1*	ENST00000338128	x	x
		0.965	1	rs2385088	A/G	G	G	ENSG00000105655	*ISYNA1*	ENST00000582811	x	x
		1	1	rs2303697	T/C	C	C	ENSG00000105655	*ISYNA1*	ENST00000582770*		x

		1	1	rs2303697	T/C	C	C	ENSG00000105655	*ISYNA1*	ENST00000582811*		x
		1	1	rs2303697	T/C	C	C	ENSG00000105655	*ISYNA1*	ENST00000577820*		x
		0.897	1	rs1043327	A/G	G	G	ENSG00000105656	*ELL*	ENST00000594635	x	x
		0.897	1	rs1043327	A/G	G	G	ENSG00000105656	*ELL*	ENST00000262809	x	x
20q11.22	rs2284378	1	1	rs6119447	A/G	G	A	ENSG00000125970	*RALY*	ENST00000375114	x	x
		1	1	rs8123521	A/C	C	A	ENSG00000125970	*RALY*	ENST00000375114	x	x
22q12.1	rs17879961	–	–	rs17879961	A/C/G	A	G	ENSG00000183765	*CHEK2*	ENST00000454252*		x

* Non-sense mediated decay transcript (Ensembl v92)
† Transcript not available at TargetScan's dataset of 3'UTRs whole-genome alignments
[1] Linkage disequilibrium, obtained from SNAP (Pilot release of 1000 Genomes Project; CEU population; $r^2 \geq 0.8$; distance limit = 500 kb)
[2] Ancestral allele, retrieved from the Ensembl database v92
[3] Minor allele, retrieved from the Ensembl database v92

Figure S1. Majority of breast cancer risk-associated SNPs lie in non-coding regions.

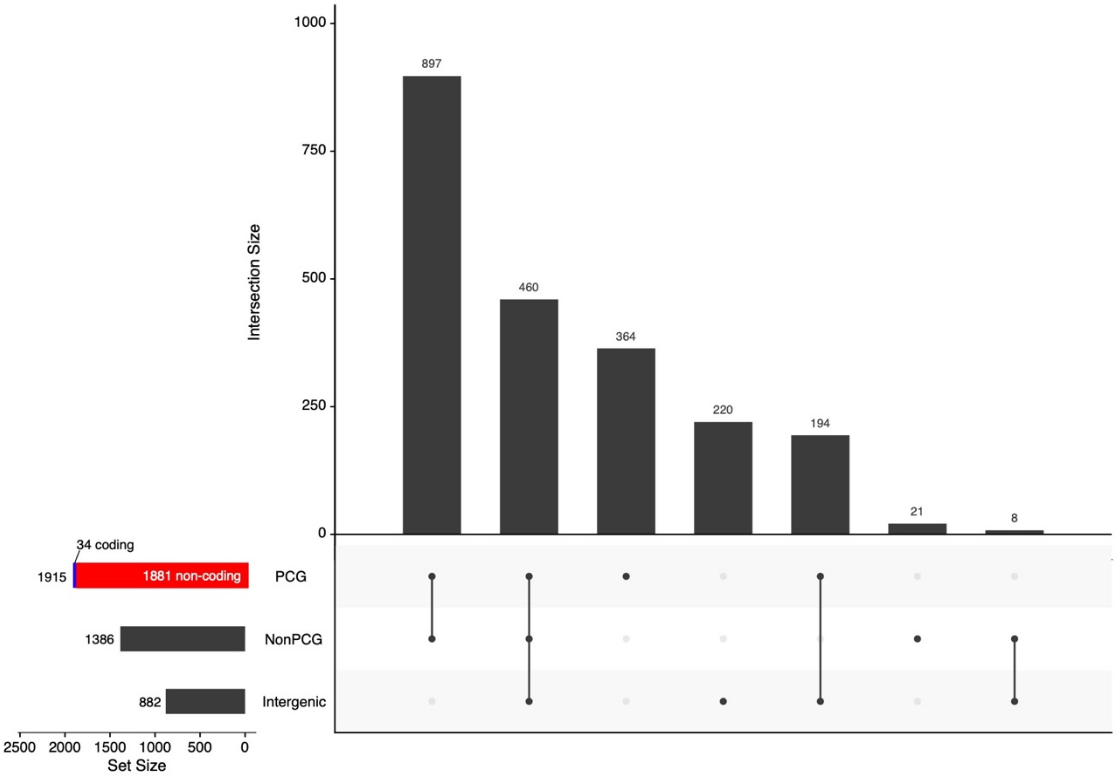

Figure S2. GTEx expression (v7) of transcripts predicted to be affected by candidate rSNPs at 19p13.11 and 1q21.1 loci in breast (mammary tissue). Panel to the left indicates total expression levels for the gene. Panel to the right indicates expression levels for all transcripts of each gene individually. Expression levels are in Transcripts per Million (TPM).

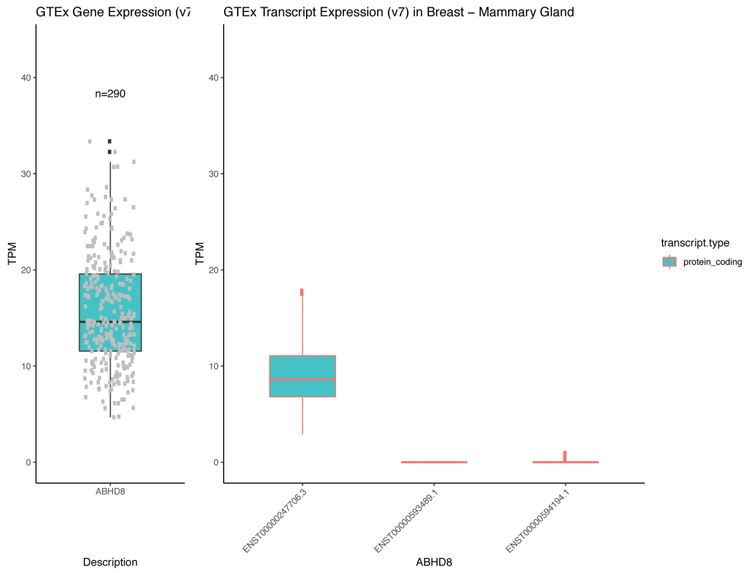

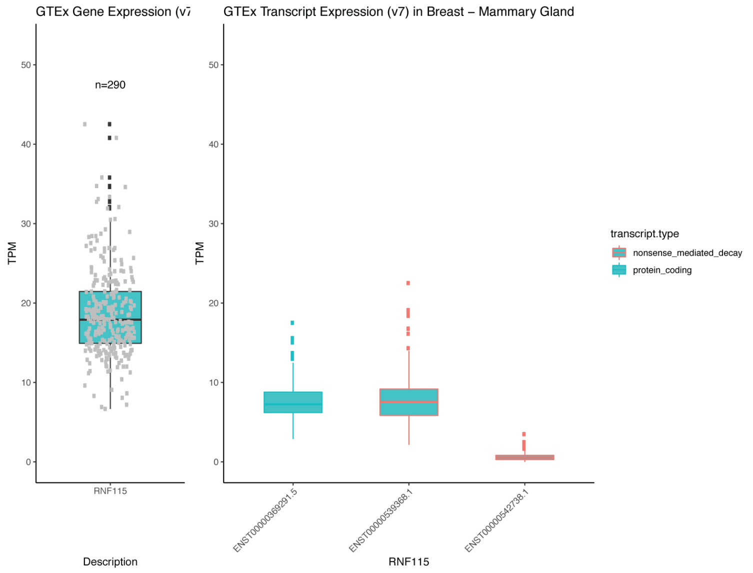

Figure S3. DAE data for *ATG10*, *CCDC170* and *ISYNA1* in breast (mammary tissue). DAE data for heterozygous individuals tested for the SNP indicated immediately below. The alleles are indicated for each SNP in the order of the AE ratio calculated (i.e. A/G corresponds to the ratio of allele A by allele G). Dashed horizontal lines indicate the threshold for DAE set at 1.5 fold difference between alleles (|$\log_2$ AE_{ratio}|= 0.58).

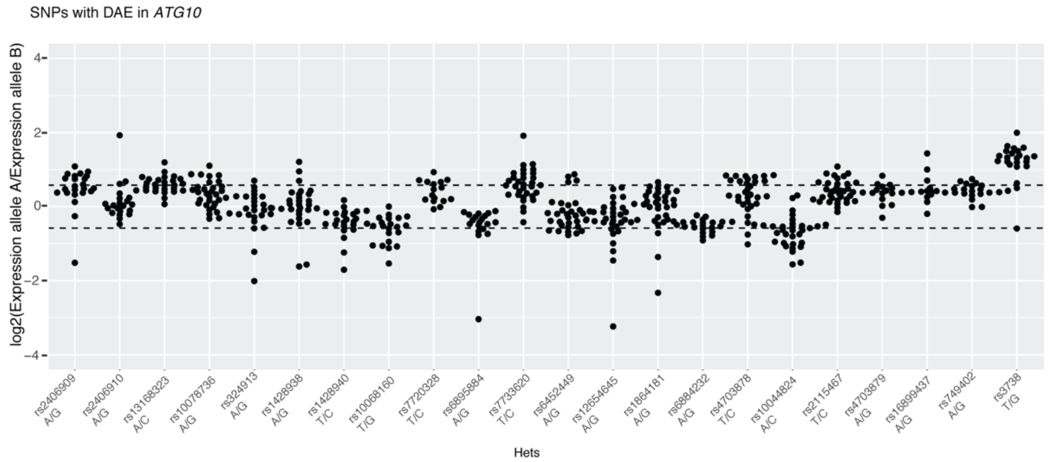

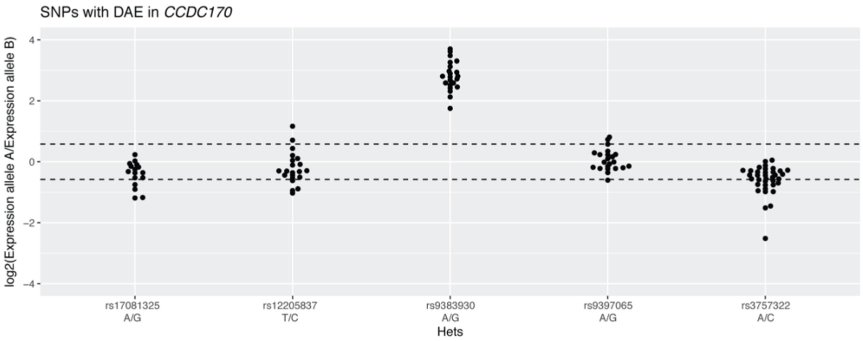

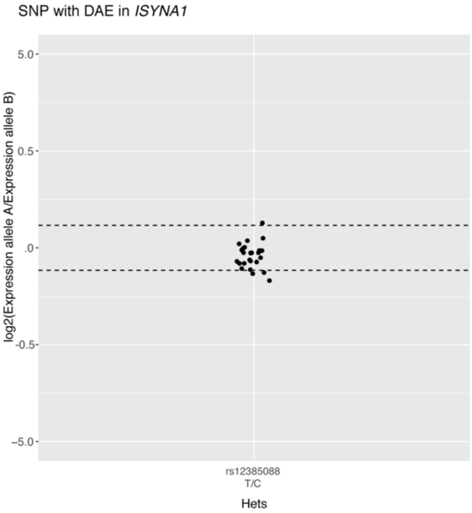

Supplementary Data Set 1 - A tab-separated text file with TargetScan's miRNA binding predictions.

Supplementary Data Set 2 - A tab-separated text file with miRanda's miRNA binding predictions.

References

1. Fehringer G, Kraft P, Pharoah PD, Eeles RA, Chatterjee N, Schumacher FR, et al. Cross-Cancer Genome-Wide Analysis of Lung, Ovary, Breast, Prostate, and Colorectal Cancer Reveals Novel Pleiotropic Associations. Cancer Res [Internet]. American Association for Cancer Research; 2016 [cited 2017 Sep 26];76:5103–14. Available from: http://www.ncbi.nlm.nih.gov/pubmed/27197191

2. Garcia-Closas M, Couch FJ, Lindstrom S, Michailidou K, Schmidt MK, Brook MN, et al. Genome-wide association studies identify four ER negative-specific breast cancer risk loci. Nat Genet. 2013;45:392–8.

3. Michailidou K, Hall P, Gonzalez-Neira A, Ghoussaini M, Dennis J, Milne RL, et al. Large-scale genotyping identifies 41 new loci associated with breast cancer risk. Nat Genet [Internet]. 2013;45:353–61, 361e1-2. Available from: http://www.nature.com/doifinder/10.1038/ng.2563

4. Han M-R, Long J, Choi J-Y, Low S-K, Kweon S-S, Zheng Y, et al. Genome-wide association study in East Asians identifies two novel breast cancer susceptibility loci. McCarthy MI, editor. Hum Mol Genet [Internet]. 2016;25:3361–71. Available from: http://dx.plos.org/10.1371/journal.pgen.1002532

5. Thomas G, Jacobs KB, Kraft P, Yeager M, Wacholder S, Cox DG, et al. A multistage genome-wide association study in breast cancer identifies two new risk alleles at 1p11.2 and 14q24.1 (RAD51L1). Nat Genet. 2009;41:579–84.

6. Michailidou K, Beesley J, Lindstrom S, Canisius S, Dennis J, Lush MJ, et al. Genome-wide association analysis of more than 120,000 individuals identifies 15 new susceptibility loci for breast cancer. Nat Genet [Internet]. 2015;47:373–80. Available from: http://www.nature.com/doifinder/10.1038/ng.3242

7. Cai Q, Zhang B, Sung H, Low SK, Kweon SS, Lu W, et al. Genome-wide association analysis in East Asians identifies breast cancer susceptibility loci at 1q32.1, 5q14.3 and 15q26.1. Nat Genet [Internet]. Nature Publishing Group; 2014;46:886–90. Available from: http://dx.doi.org/10.1038/ng.3041

8. Kim H cheol, Lee JY, Sung H, Choi JY, Park SK, Lee KM, et al. A genome-wide association study identifies a breast cancer risk variant in ERBB4 at 2q34: Results from the Seoul Breast Cancer Study. Breast Cancer Res. 2012;14.

9. Stacey SN, Manolescu A, Sulem P, Rafnar T, Gudmundsson J, Gudjonsson SA, et al. Common variants on chromosomes 2q35 and 16q12 confer susceptibility to estrogen receptor-positive breast cancer. Nat Genet. 2007;39:865–9.

10. Thomas LF, Sætrom P. Single Nucleotide Polymorphisms Can Create Alternative Polyadenylation Signals and Affect Gene Expression through Loss of MicroRNA-Regulation. PLoS Comput Biol. 2012;8.

11. Fletcher O, Johnson N, Orr N, Hosking FJ, Gibson LJ, Walker K, et al. Novel Breast Cancer Susceptibility Locus at 9q31.2: Results of a Genome-Wide Association Study. JNCI J Natl Cancer Inst [Internet]. Oxford University Press; 2011 [cited 2017 Sep 30];103:425–35. Available from: https://academic.oup.com/jnci/article-lookup/doi/10.1093/jnci/djq563

12. Turnbull C, Ahmed S, Morrison J, Pernet D, Renwick A, Maranian M, et al. Genome-wide association study identifies five new breast cancer susceptibility loci. Nat Genet. 2010;42:504–7.

13. Ahsan H, Halpern J, Kibriya MG, Pierce BL, Tong L, Gamazon E, et al. A genome-wide association study of early-onset breast cancer identifies PFKM as a novel breast cancer gene and supports a common genetic spectrum for breast cancer at any age. Cancer Epidemiol Biomarkers Prev. 2014;23:658–69.

14. Haiman CA, Chen GK, Vachon CM, Canzian F, Dunning A, Millikan RC, et al. A common variant at the TERT-CLPTM1L locus is associated with estrogen receptorĝ€"negative breast cancer. Nat Genet. 2011;43:1210–4.

15. Couch FJ, Kuchenbaecker KB, Michailidou K, Mendoza-Fandino GA, Nord S, Lilyquist J, et al. Identification of four novel susceptibility loci for oestrogen receptor negative breast cancer. Nat Commun [Internet]. 2016;7:11375. Available from: http://www.nature.com/doifinder/10.1038/ncomms11375

16. Easton DF, Pooley KA, Dunning AM, Pharoah PDP, Thompson D, Ballinger DG, et al. Genome-wide association study identifies novel breast cancer susceptibility loci. Nature [Internet]. 2007;447:1087–93. Available from: http://www.nature.com/doifinder/10.1038/nature05887

17. Gold B, Kirchhoff T, Stefanov S, Lautenberger J, Viale A, Garber J, et al. Genome-wide association study provides evidence for a breast cancer risk locus at 6q22.33. Proc Natl Acad Sci U S A [Internet]. 2008;105:4340–5. Available from: http://www.pubmedcentral.nih.gov/articlerender.fcgi?artid=2393811&tool=pmcentrez&rendertype=abstract

18. Long J, Cai Q, Sung H, Shi J, Zhang B, Choi JY, et al. Genome-wide association study in East Asians identifies novel susceptibility loci for breast cancer. PLoS Genet. 2012;8.

19. Zheng W, Long J, Gao Y-T, Li C, Zheng Y, Xiang Y-B, et al. Genome-wide association study identifies a new breast cancer susceptibility locus at 6q25.1. Nat Genet [Internet]. 2009;41:324–8. Available from: http://www.nature.com/doifinder/10.1038/ng.318

20. Couch FJ, Wang X, McGuffog L, Lee AA, Olswold C, Kuchenbaecker KB, et al. Genome-wide association study in BRCA1 mutation carriers identifies novel loci associated with breast and ovarian cancer risk. PLoS Genet [Internet]. 2013;9:e1003212. Available from: http://www.ncbi.nlm.nih.gov/entrez/query.fcgi?cmd=Retrieve&db=PubMed&dopt=Citation&list_uids=23544013%5Cnhttp://www.pubmedcentral.nih.gov/articlerender.fcgi?artid=3609646&tool=pmcentrez&rendertype=abstract

21. Fejerman L, Ahmadiyeh N, Hu D, Huntsman S, Beckman KB, Caswell JL, et al. Genome-wide association study of breast cancer in Latinas identifies novel protective variants on 6q25. Nat Commun. 2014;5.

22. Siddiq A, Couch FJ, Chen GK, Lindström S, Eccles D, Millikan RC, et al. A meta-analysis of genome-wide association studies of breast cancer identifies two novel susceptibility loci at 6q14 and 20q11. Hum Mol Genet. 2012;21:5373–84.

23. Michailidou K, Lindström S, Dennis J, Beesley J, Hui S, Kar S, et al. Association analysis identifies 65 new breast cancer risk loci. Nature. 2017;551:92–4.

24. Cai Q, Long J, Lu W, Qu S, Wen W, Kang D, et al. Genome-wide association study identifies breast cancer risk variant at 10q21.2: Results from the asia breast cancer consortium. Hum Mol Genet. 2011;20:4991–9.

25. Low S-K, Takahashi A, Ashikawa K, Inazawa J, Miki Y, Kubo M, et al. Genome-Wide Association Study of Breast Cancer in the Japanese Population. PLoS One [Internet]. 2013;8:e76463. Available from: http://dx.plos.org/10.1371/journal.pone.0076463

26. Gaudet MM, Kirchhoff T, Green T, Vijai J, Korn JM, Guiducci C, et al. Common genetic variants and modification of penetrance of BRCA2-associated breast cancer. PLoS Genet. 2010;6:1–12.

27. Li J, Humphreys K, Heikkinen T, Aittomäki K, Blomqvist C, Pharoah PDP, et al. A combined analysis of genome-wide association studies in breast cancer. Breast Cancer Res Treat. 2011;126:717–27.

28. Hunter DJ, Kraft P, Jacobs KB, Cox DG, Yeager M, Hankinson SE, et al. A genome-wide association study identifies alleles in FGFR2 associated with risk of sporadic postmenopausal breast cancer. Nat Genet. 2007;39:870–4.

29. Purrington KS, Slager S, Eccles D, Yannoukakos D, Fasching PA, Miron P, et al. Genome-wide association study identifies 25 known breast cancer susceptibility loci as risk factors for triple-negative breast cancer. Carcinogenesis. 2014;35:1012–9.

30. Orr N, Lemnrau A, Cooke R, Fletcher O, Tomczyk K, Jones M, et al. Genome-wide association study identifies a common variant in RAD51B associated with male breast cancer risk. Nat Genet [Internet]. Nature Publishing Group; 2012;44:1182–4. Available from: http://dx.doi.org/10.1038/ng.2417

31. Long J, Cai Q, Shu X-O, Qu S, Li C, Zheng Y, et al. Identification of a functional genetic variant at 16q12.1 for breast cancer risk: results from the Asia Breast Cancer Consortium. PLoS Genet [Internet]. 2010;6:e1001002. Available from: http://www.ncbi.nlm.nih.gov/pubmed/20585626%5Cnhttp://www.pubmedcentral.nih.gov/articlerender.fcgi?artid=PMC2891809

32. Antoniou AC, Wang X, Fredericksen ZS, McGuffog L, Tarrell R, Sinilnikova OM, et al. A locus on 19p13 modifies risk of breast cancer in BRCA1 mutation carriers and is associated with hormone receptor-negative breast cancer in the general population. Nat Genet. 2010;42:885–92.

33. Agarwal V, Bell GW, Nam J-W, Bartel DP. Predicting effective microRNA target sites in mammalian mRNAs. Elife [Internet]. 2015;4:1–38. Available from: http://elifesciences.org/lookup/doi/10.7554/eLife.05005

34. Enright AJ, John B, Gaul U, Tuschl T, Sander C, Marks DS. MicroRNA targets in Drosophila. Genome Biol [Internet]. 2003;5:R1. Available from: http://genomebiology.biomedcentral.com/articles/10.1186/gb-2003-5-1-r1

35. John B, Enright AJ, Aravin A, Tuschl T, Sander C, Marks DS. Human MicroRNA Targets. James C. Carrington, editor. PLoS Biol [Internet]. 2004;2:e363. Available from: http://www.ncbi.nlm.nih.gov/pubmed/15502875

36. Krek A, Grün D, Poy MN, Wolf R, Rosenberg L, Epstein EJ, et al. Combinatorial microRNA target predictions. Nat Genet [Internet]. 2005;37:495–500. Available from: http://www.ncbi.nlm.nih.gov/pubmed/15806104

37. Reczko M, Maragkakis M, Alexiou P, Grosse I, Hatzigeorgiou AG. Functional microRNA targets in protein coding sequences. Bioinformatics. 2012;28:771–6.

38. Paraskevopoulou MD, Georgakilas G, Kostoulas N, Vlachos IS, Vergoulis T, Reczko M, et al. DIANA-microT web server v5.0: service integration into miRNA functional analysis workflows. Nucleic Acids Res [Internet]. Oxford University Press; 2013 [cited 2017 Aug 29];41:W169–73. Available from: https://academic.oup.com/nar/article-lookup/doi/10.1093/nar/gkt393

39. Krüger J, Rehmsmeier M. RNAhybrid: MicroRNA target prediction easy, fast and flexible. Nucleic Acids Res. 2006;34:451–4.

40. Rehmsmeier M, Steffen P, Höchsmann M, Giegerich R, Ho M. Fast and effective prediction of microRNA / target duplexes. Spring. 2004;1507–17.

41. Kertesz M, Iovino N, Unnerstall U, Gaul U, Segal E. The role of site accessibility in microRNA target recognition. Nat Genet [Internet]. 2007;39:1278–84. Available from: /Users/yurikoharigaya/Documents/ReadCube Media/kertesz2007.pdf%5Cnhttp://dx.doi.org/10.1038/ng2135

42. Hsu J, Chiu C-M, Hsu S-D, Huang W-Y, Chien C-H, Lee T-Y, et al. miRTar: an integrated system for identifying miRNA-target interactions in human. BMC Bioinformatics [Internet]. 2011;12:300. Available from: http://bmcbioinformatics.biomedcentral.com/articles/10.1186/1471-2105-12-300
